# The evaluation of anoxia responsive E2F DNA binding activity in the red eared slider turtle, *Trachemys scripta elegans*

**DOI:** 10.7717/peerj.4755

**Published:** 2018-05-11

**Authors:** Kyle K. Biggar, Kenneth B. Storey

**Affiliations:** 1 Institute of Biochemistry, Carleton University, Ottawa, Ontario, Canada; 2 Institute of Biochemistry, Depts of Biology and Chemistry, Carleton University, Ottawa, Ontario, Canada

**Keywords:** Hypometablism, Metabolic rate depression, Cell cycle, Protein complex

## Abstract

In many cases, the DNA-binding activity of a transcription factor does not change, while its transcriptional activity is greatly influenced by the make-up of bound proteins. In this study, we assessed the protein composition and DNA-binding ability of the E2F transcription factor complex to provide insight into cell cycle control in an anoxia tolerant turtle through the use of a modified ELISA protocol. This modification also permits the use of custom DNA probes that are tailored to a specific DNA binding region, introducing the ability to design capture probes for non-model organisms. Through the use of EMSA and ELISA DNA binding assays, we have successfully determined the *in vitro* DNA binding activity and complex dynamics of the Rb/E2F cell cycle regulatory mechanisms in an anoxic turtle, *Trachemys scripta elegans*. Repressive cell cycle proteins (E2F4, Rb, HDAC4 and Suv39H1) were found to significantly increase at E2F DNA-binding sites upon anoxic exposure in anoxic turtle liver. The lack of p130 involvement in the E2F DNA-bound complex indicates that anoxic turtle liver may maintain G_1_ arrest for the duration of stress survival.

## Introduction

Heterochromatin is composed of genomic DNA tightly packed by histones and non-histone proteins (Lu & Gilbert, 2007; Ferreira et al., 2001). Dynamic changes in chromatin structure prevent the access of transcription factors, such as E2F, to nucleosomal DNA. At least two primary mechanisms can be used by the cell to remodel chromatin structure. One mechanism involves changing the location and conformation of the nucleosomes through the use of ATP-dependent protein complexes such as the SWItch/Sucrose Non Fermentable complex (SWI/SNF) (Zhang & Dean, 2001; Kingston & Narlikar, 1999; Muchardt & Yaniv, 1999; Tyler & Kadonaga, 1999; Van den Heuvel & Dyson, 2008). The second mechanism involves covalent modifications of histone N-terminal tails that protrude from the chromatin structure (Ferreira et al., 2001; Trojer et al., 2007; Wu, Connelly & Biggar, 2017). Studies examining the role of the retinoblastoma (Rb)/E2F mediated cell cycle arrest have identified key Rb interactions with chromatin remodeling factors. Importantly, the particular associations between Rb and chromatin remodeling factors have been found to be dependent on the type of cell cycle exit (Lu & Gilbert, 2007).

The annotation of protein complexes is often coupled with 2-dimensional gel electrophoresis or mass spectrometry (Free, Hazelwood & Sibley, 2009); however, this technique falls short in assessing the DNA binding activity or separating transcriptionally active from inactive transcription factor protein complexes. This information is critical when studying protein complexes that consist of multiple members and whose composition changes dynamically depending on the degree and type of DNA binding activity (Robertson et al., 2000; Huang & Hearing, 1989; Litovchick et al., 2007; Guardavaccaro & Pagano, 2006). In many cases, the presence of a transcription factor at a gene promoter may not change while its transcriptional activity is greatly influenced by the make-up of binding partners. This is true for the G_1_/S-transition transcription factor, E2F, where transcriptional activity and cell cycle control is dictated by the dynamic binding of several members of the Rb family members and chromatin remodeling proteins.

Our study utilizes a novel modification to an enzyme-linked immunosorbent assay (ELISA) to assess the protein composition and DNA-binding ability of transcription factor complexes. The use of custom DNA probes provides a benefit over traditional co-IPs which use capture antibodies, commonly derived from well-characterized mammalian animal models to purify protein complexes. This type of complex purification results in a high degree of false-positives in non-mammalian organisms, often due to the use of mammalian-derived antibodies and a high degree of non-specific cross-reactivity. However, DNA binding sites are highly specific for a particular transcription factor and are often conserved throughout much of evolution (Van den Heuvel & Dyson, 2008).

**Figure 1:**
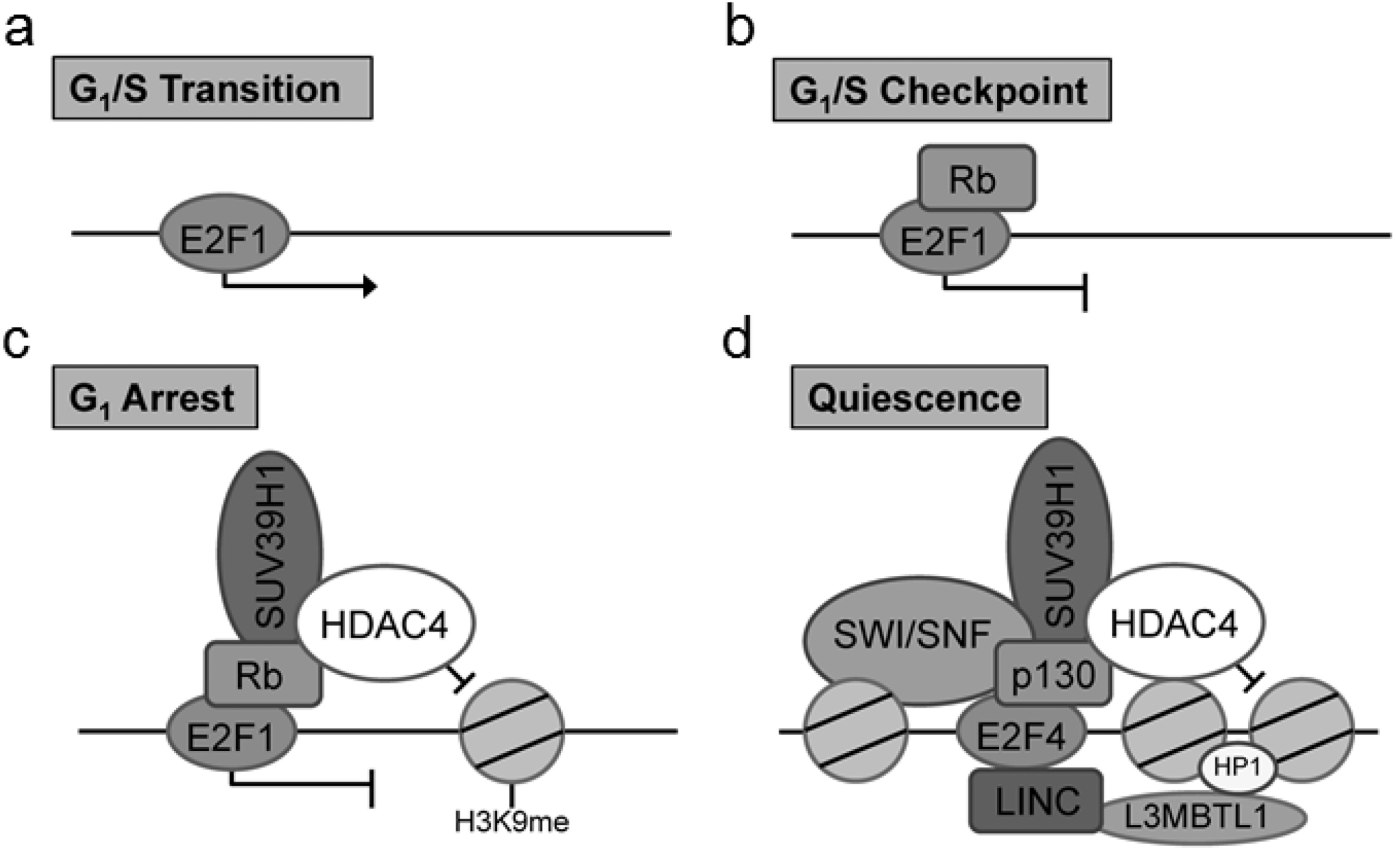
Complex composition of E2F at (A) G_1_/S transition, (B) G_1_/S checkpoint complex between E2F1 and Rb, (C) G_1_ cell cycle arrest marked by a complex with E2F1, Rb, SUV39H1, and HDAC4, and (D) cellular quiescence indicated through a complex between E2F4, p130, SUV39H1, HDAC4, L3MBTL1, HP1, and SWI/SNF. These unique E2F complexes highlight critical stages of the cell cycle and cell cycle arrest mediated through the G_1_ transition.

Of particular interest is the identification of the cell cycle regulatory protein interactions between the E2F and retinoblastoma (Rb) proteins which function together to control the cell cycle. Previous co-IP studies examining the role of Rb/E2F mediated cell cycle exit have identified key Rb interactions with chromatin remodeling factors including histone deacetylase family member 4 (HDAC4) and suppressor of variegation 3–9 homolog 1 (Suv39H1) (Lu & Gilbert, 2007). Critically, the particular associations between Rb and chromatin remodeling factors have been found to be dependent on the type of cell cycle exit (Lu & Gilbert, 2007). These chromatin modifications are well cited in the literature for the G_1_/S transition, G_1_/S checkpoint, G_1_ arrest and quiescence (G_0_) (Sadasivam & DeCaprio, 2013). There are notable modifications to the composition of the DNA-bound E2F complex in cells entering a pause of the cell cycle (G_1_ arrest) and those entering a prolonged exit (quiescence) (Fig. 1). To evaluate the usefulness of the proposed technique, we have used this method to explore Rb/E2F complex formation and DNA binding activity during prolonged anoxia in a tolerant turtle, the red-eared slider (*Trachemys scripta elegans*). The red-eared slider turtle is a well-documented facultative anaerobe that enters into a suspended state of metabolic rate depression during periods of extended oxygen deprivation (Storey & Storey, 2004; Storey & Storey, 2007; Biggar & Storey, 2009). In these oxygen-deprived states, the turtle halts unnecessary ATP utilization by reducing energy consumption of non-essential cellular processes until normoxia can be restored; one such non-essential cellular process previously proposed to be under active regulatory control during anoxia is the progression of the cell cycle (Biggar & Storey, 2009; Roufayel, Biggar & Storey, 2011; Biggar & Storey, 2012; Zhang, Biggar & Storey, 2013).

The purpose of this study was to evaluate the DNA binding activity and the protein complex associated with the E2F1 and E2F4 transcription factors in the anoxia tolerant turtle. To facilitate this study, we developed a method to identify protein binding partners in transcription factors that are actively binding to DNA *in vitro*. This method accomplishes this task by separating inactive and active transcriptional complexes through the use of conserved DNA binding elements. The use of DNA binding elements also allows this protocol to be adapted to new or uncharacterized model animals where traditional mammalian antibody-based precipitation cannot be utilized.

## Materials and Methods

### Animals

Adult female *T. scripa elegans* were purchased from Carolina Biological (Burlington, NC, USA). Animal handling and anoxic experiments were as described previously (Krivoruchko & Storey, 2010). Turtles had been previously held at ∼11 °C and were initially placed at this temperature in a large holding tank containing dechlorinated water. The temperature was then lowered to ∼4 °C over 1 week and then animals were acclimated at 4 ± 1 °C for 2 weeks before use. Aerobic control turtles were sampled from this condition. To impose anoxia, other turtles were moved into containers filled with dechlorinated water at 4 °C that had been previously bubbled with 100% nitrogen gas for at least 6 h beforehand. After turtles were placed in a tank, a wire mesh was fitted ∼10 cm below the water line so that turtles could not surface during the anoxic episode; a low level of nitrogen bubbling continued throughout. Animals were subjected to either 5 or 20 hr of anoxic submergence and were then rapidly removed and sampled. All turtles survived the experimental treatments. Animals were killed by decapitation and tissues were rapidly dissected out, immediately frozen in liquid nitrogen and then transferred to −80 °C for long-term storage. All animals were cared for in accordance with the guidelines of the Canadian Council on Animal Care and all experimental procedures had the prior approval of the Carleton University Animal Care Committee (IACUC approval #B09-20).

### Preparation of nuclear extracts

Nuclear extracts were prepared using a slight modification of the method described by Dignam, Lebovitz & Roeder (1983). Briefly, samples of frozen liver tissue (∼1 g) were homogenized in 1 mL of homogenization buffer (10 mM HEPES, pH 7.9, 10 mM KCl, 10 mM EDTA) with the addition of 10 µL of 100 mM dithiothreitol (DTT) and 10 µL protease inhibitor cocktail. Samples were centrifuged at 10,000 rpm for 10 min at 4 °C and the supernatant (cytoplasmic extract) was removed. The pellet was resuspended in 147 µL of extraction buffer (20 mM HEPES, pH 7.9, 400 mM NaCl, 1 mM EDTA, 10% v:v glycerol) with 1.5 µL of 100 mM DTT and 1.5 µL of protease inhibitor cocktail added. The tubes were capped, put in ice on a rocking platform for 1 h, and then centrifuged at 10,000× g for 10 min at 4 °C. The supernatant (nuclear extract) was collected. The integrity of the nuclei was confirmed by immunoblotting of cytoplasmic and nuclear fractions and probing with a histone H3 antibody (Cell Signaling; Cat# 9715).

### Enzyme-linked immunosorbent assays

Aliquots containing equal amounts of nuclear-isolated protein from each sample (16–32 µg/well) were then used to assess the amount of binding by E2F1 and E2F4 to the E2F DNA binding sequence. The sequence of the biotin-conjugated probe was 5′- Biotin-ATTTAAGTTTCGCGCCCTTTCTCAA -3′, where the E2F DNA binding element used in this study is underlined. Both sense and anti-sense oligonucleotides were purchased from Sigma Genosys, diluted to 500 µM using sterile H_2_O and subsequently mixed 1:1 (v/v) for a total volume of 20 µl. Probes were then placed in a thermocycler for 10 min at 95 °C and the temperature of the block was allowed to passively cool to room temperature (RT). A 40 pmol aliquot of double-stranded, biotinylated probe in 50 µL of phosphate buffered saline (PBS; 137 mM NaCl, 2.7 mM KCl, 10 mM Na_2_HPO_4_, 2 mM KH_2_PO_4_, pH 7.4) was then added to each well of a streptavidin-coated microplate (VWR; Cat# 732-2681). The plate was then incubated at RT for 1 hr and washed twice with wash buffer (0.1% Tween-20 in PBS) and once with PBS. Aliquots of 16–32 µg protein were combined with 50 µL of 1× protein binding buffer (10 mM Hepes, pH 7.9, 50 mM KCl, 0.5 mM EDTA, 3 mM MgCl_2_, 10% glycerol, 0.5 mg/mL BSA, 0.05% NP-40, 1–2 µg salmon sperm DNA (BioShop; Cat# DNA004.1), 0.5 mM DTT, and 80 mM NaCl) and then added to the wells. Samples were incubated at RT with mild agitation for 60–90 min followed by four washes with wash buffer. A 60 µL aliquot of the appropriate antibody, diluted in PBS, was added to the wells and the plate was incubated for 1–1.5 hr. The plate was washed as above and then 60 µL of appropriate IgG-HRP secondary antibody (BioShop) diluted 1:2,000 in PBS was added and the plate was incubated for 1 hr at RT. Following incubation with secondary antibody, the plate was washed as above and 60 µL of TMB (tetramethylbenzidine) was added (Bioshop; Cat# TMB333). Optimization of protein binding conditions is listed in the Supplemental Information. Once colour had developed, the reaction was stopped by the addition of 1 M HCl and optical density was read at 450 nm with a reference wavelength of 655 nm using a Multiskan Spectrum (Thermo Labsystems, Beverly, MA, USA). For competition assays, a 100-fold molar excess of unlabeled probe was pre-incubated for 20 min at room temperature with nuclear extracts before the labeled probe.

### Antibodies

All primary antibodies were purchased from commercial suppliers. Rabbit anti-E2F1 (Cat# sc-193), rabbit anti-E2F4 (Cat# sc-512), goat anti-phospho-p130 (Thr986) (Cat# sc-16299), goat anti-Suv39H1 (Cat# sc-13608) and rabbit anti-XBP1 (Cat# sc-7160) polyclonal antibodies were purchased from Santa Cruz Biotechnology (Santa Cruz, CA, USA); rabbit anti-HDAC4 (Cat# Ab-632) polyclonal antibody was purchased from GenScript (Piscataway, NJ, USA); mouse anti-Rb (Cat# 9309S) was purchased from Cell Signal Technology (Danvers, MA, USA). All primary antibodies were raised against mammalian antigens and were diluted in PBS according to the manufacturer’s specifications.

### Electrophoretic mobility shift assay

Electrophoretic mobility shift assays were performed by using the LightShift^®^ Chemiluminescent EMSA Kit (Pierce, Waltham, MA) with the same double-stranded 5′ biotin-labeled oligonucleotide probe as previously listed. For each binding reaction (10 µl), a total of 10 ng biotin-labeled probe was combined with 5 µg nuclear extract, 1 µg poly(dI–dC), and 1× Binding buffer (10 mM Tris, pH 7.8, 50 mM NaCl, 1 mM EDTA, 5% glycerol). A commercially available positive control was utilized to determine the success of cross-reactivity (Cat# AY1010P; Panomics, Waltham, MA, USA). Protein–DNA complexes were analyzed by electrophoresis on non-denaturing 6.0% polyacrylamide gels in 0.5× TBE. Gels were transferred using a standard wet-transfer electroblotting procedure for 30 min at 300 mA and transferred to Pall Biodyne B nylon membranes (VWR International, Radnor, PA, USA). Membranes were visualized using streptavidin-HRP and developed using enhanced chemiluminescence reagents.

### Data and statistics

Immunoblots were imaged using the ChemiGenius Bio-imaging System (Syngene, Cambridge, UK). ELISA data were obtained from a Thermo Multiscan Spectrum reader using a wavelength of 450 nm and a reference at 655 nm. To normalize ELISA results, the OD from the negative control was subtracted from the OD of each experimental sample. Mean normalized band densities ±SEM were then calculated for control and anoxic samples. Data were analyzed using ANOVA and significant differences between control and anoxic values were identified using the Dunnett’s posthoc test.

## Results

### Electromobility shift assay

**Figure 2:**
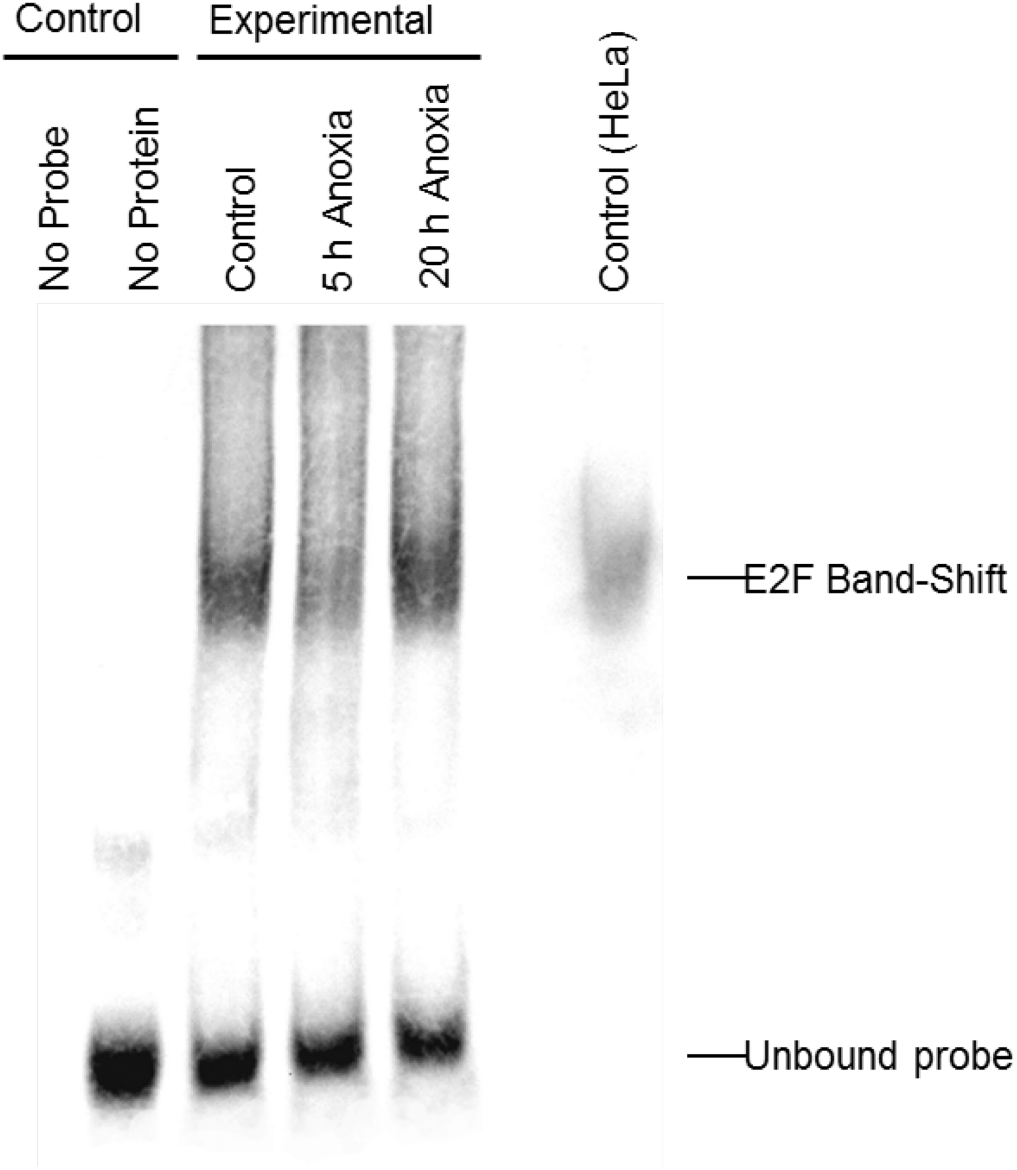
Electromobility shift assay (EMSA) for the E2F family of transcription factors. Distinct bands, as detected with streptavidin HRP, is indicative of E2F binding as detected in control, 5 h and 20 h anoxic turtle liver tissues, as well as the positive control. Negative control lanes include (1) turtle liver proteins without DNA probe and (2) DNA probe without any protein. Bands at the bottom of the gel are indicative of single (incompletely annealed) and double stranded biotinylated DNA probe.

In order to determine whether E2F could bind the DNA probes utilized in the ELISA experiments, the relative qualitative level of E2F binding to DNA was visualized in extracts from control and anoxic liver of *T. scripta elegans*. The interaction between E2F and the promoter sequence to which it binds was investigated using an electrophoretic mobility shift assay with biotin-labeled oligonucleotides containing the E2F binding site (5′-TTTCGCGC-3′). Figure 2 shows that the biotin-labeled probes bound to the E2F protein from control (lane 3), 5 hr anoxic (lane 4) and 20 hr anoxic (lane 5) liver tissues; lane 7 shows commercial E2F probes incubated together with nuclear HeLa protein lysate to serve as a positive control. Negative controls included protein without biotin-probes (lane 1) as well as biotin-probes without protein (lane 2). The relative DNA-binding of E2F binding between stresses was not determined as several family members (all of the similar molecular weight) as all E2F family members (1–7) bind the same DNA sequence.

### Effect of nuclear protein concentration on binding

**Figure 3:**
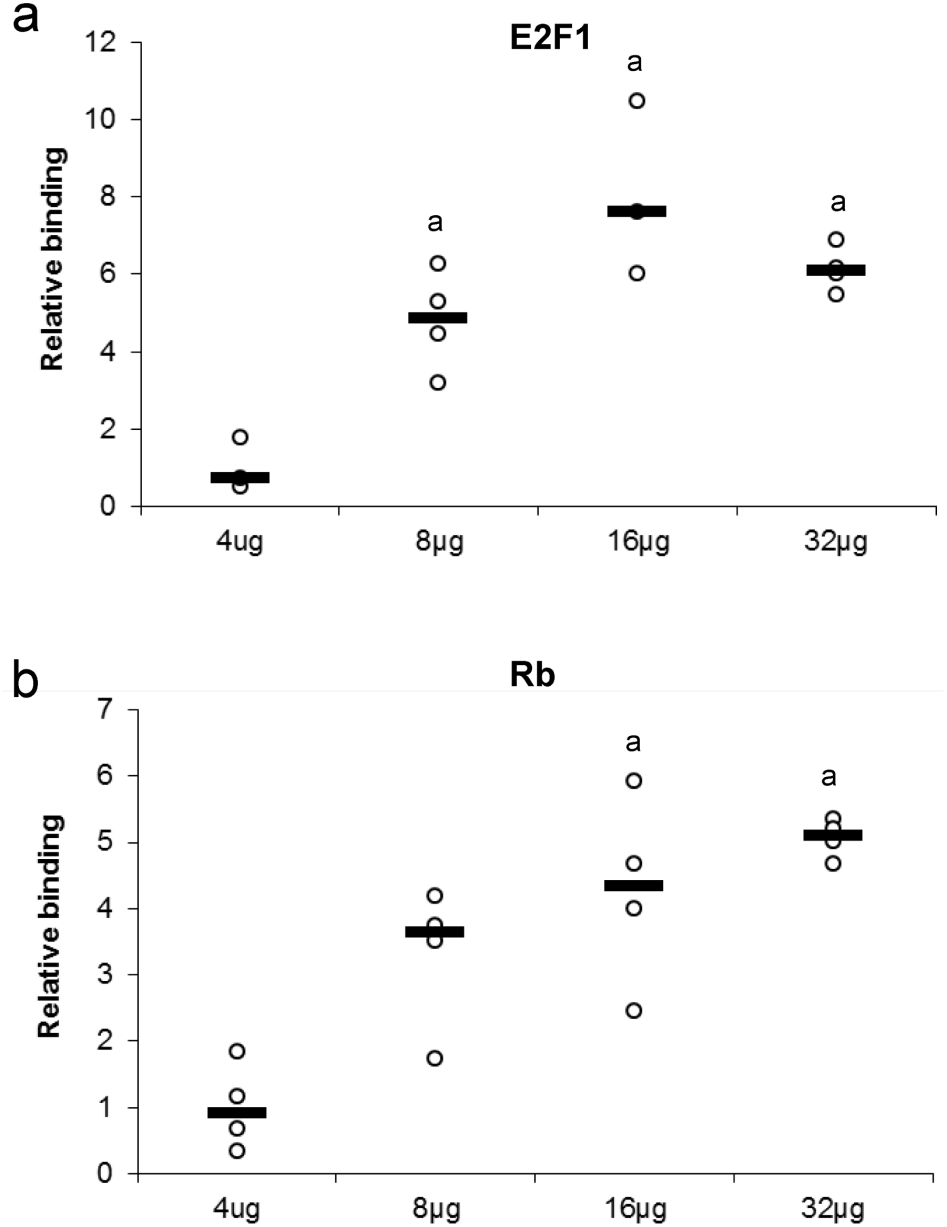
Effect of protein concentration on the relative binding of (A) E2F1 and (B) Rb in liver from control *T. scripta elegans*. The histogram shows normalized binding levels for various total protein amounts and its influence on E2F-1 and Rb binding; bold lines represent median values (*n* = 3–4 independent trials on tissue from different animals). An ‘a’ indicates significant differences from the 4 µg total protein (*P* < 0.05).

Several concentrations of nuclear protein extract were used to detect either E2F or Rb binding. This experiment tests for the ability of a protein to bind or be detected in the presence of high or low nuclear protein concentration (Fig. 3). Maximum protein binding was demonstrated between 8–32 µg of total nuclear protein for E2F1 when compared to 4 µg total nuclear protein (*P* < 0.05). Binding of Rb displayed a dose-response from 4 µg to 16 µg of total nuclear protein, where the significant and maximal binding was achieved (16–32 µg) (*P* < 0.05).

### Probe competition

**Figure 4:**
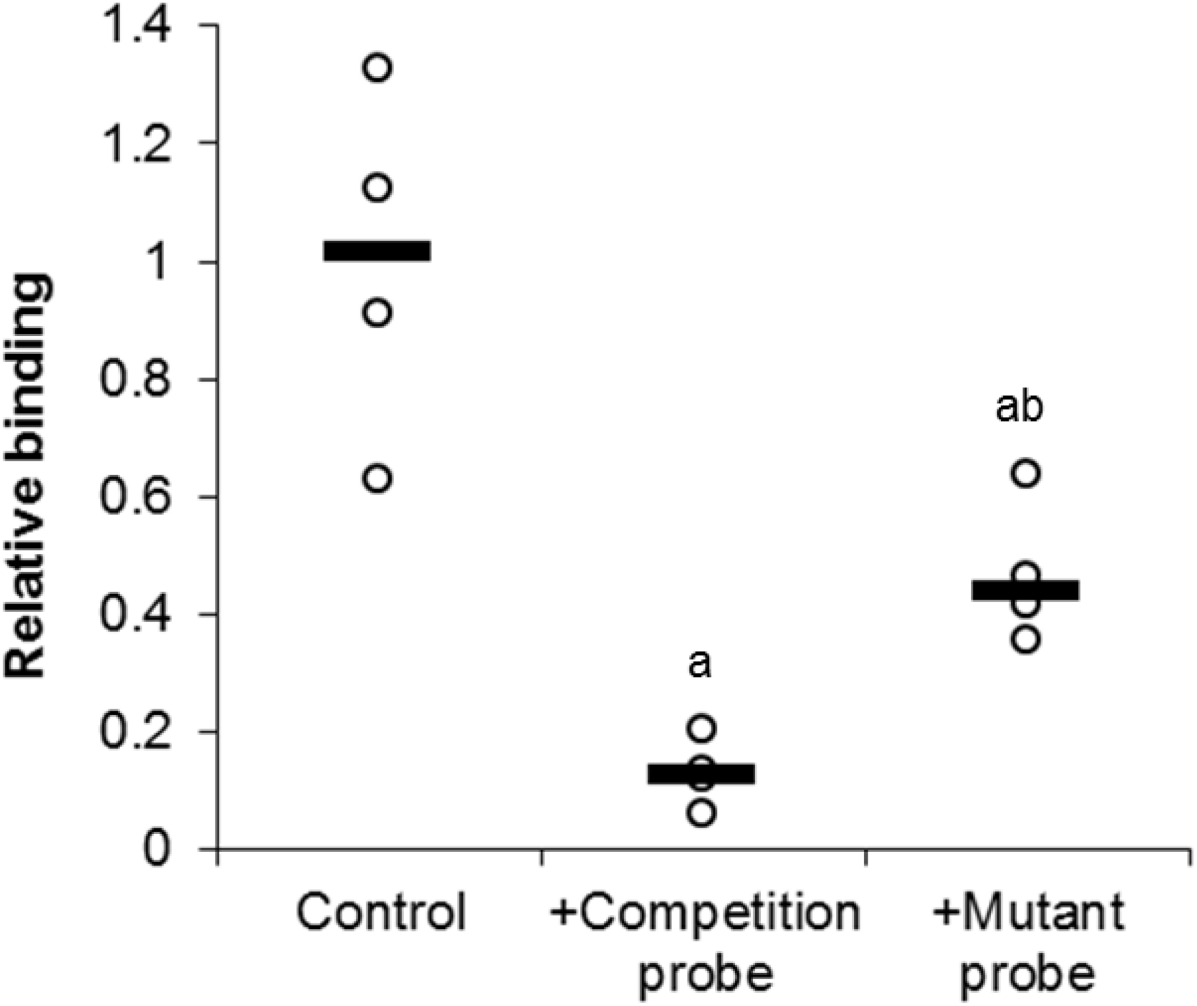
Effect of unlabeled DNA probe competition on the relative binding of E2F1 in liver from control *T. scripta elegans*. Either unlabeled DNA probe (WT) or an unlabeled DNA probe with a mutated DNA binding site (MT) was incubated with total protein in the presence of biotin-labeled probe. Reduced binding indicates successful competition between E2F1 probe binding, indicating probe specificity. The histogram shows normalized binding levels for E2F1; bold lines represent median values (*n* = 3–4 independent trials on tissue from different animals). Significant differences from the standard condition are indicated by ‘a’ (*P* < 0.05).

In order to investigate the specific binding of E2F1 to its DNA binding sequence, a competition assay was used (Fig. 4). Upon the addition of 100-molar excess non-labeled probe, the detection of E2F1 decreased to 13 ± 3% of standard conditions (*P* < 0.05). However, the addition of the 100-molar unlabeled mutated probe (5′–ATTTAAGTT**G**CG**A**GG CCTTTCTCAA-3′) only decreased the detection of E2F1 to 47 ± 6% of standard conditions (*P* < 0.05). This result indicates the specific binding of E2F1 to its DNA binding sequence and that mutation of this sequence diminishes the ability of E2F1 to bind.

### DNA binding activity and complex formation

**Figure 5:**
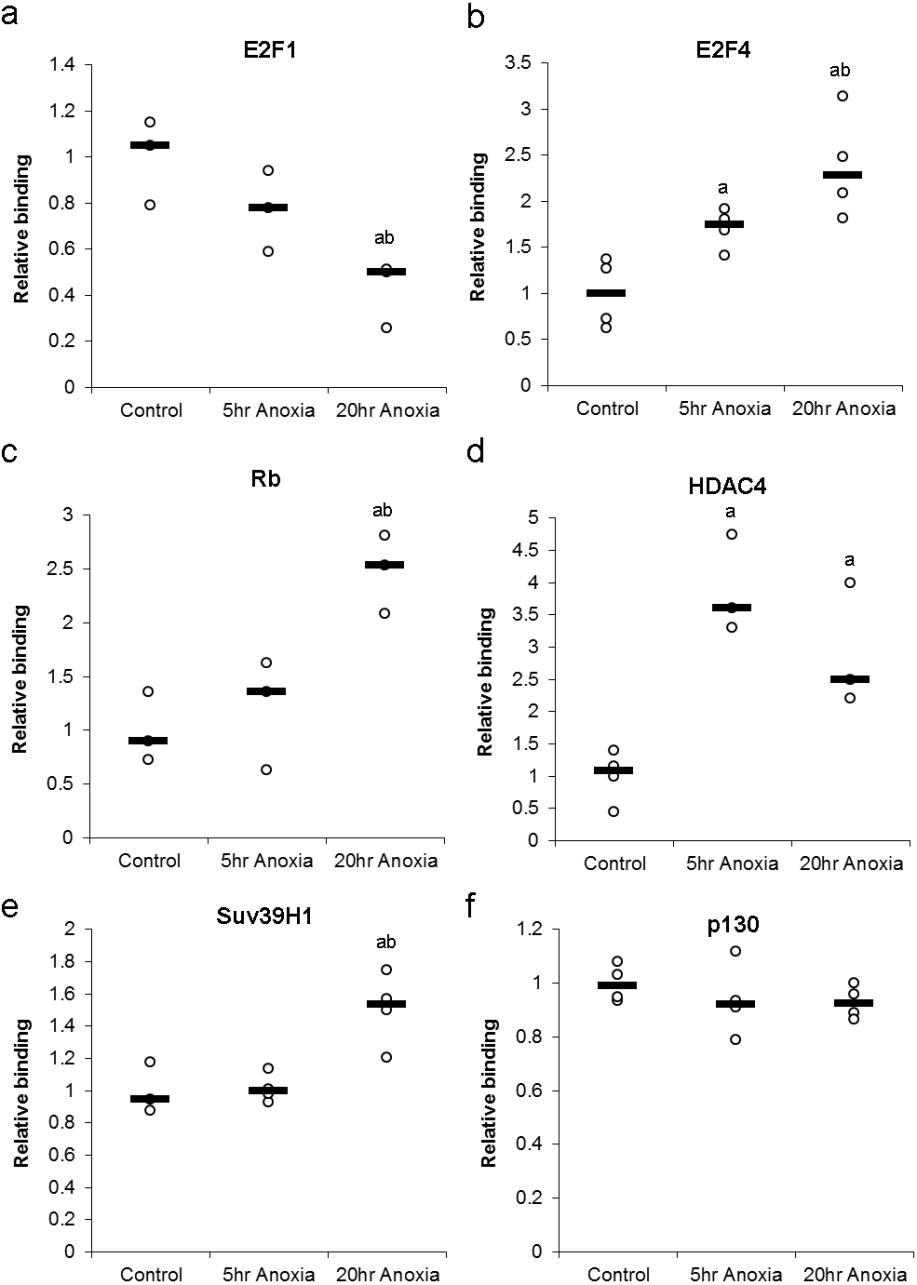
Effect of 5 and 20 h of anoxic submergence on the DNA-binding activity of (A) E2F1 and (B) E2F4 proteins, as well as the relative levels of E2F-bound (C) Rb protein, (D) HDAC4 protein, (E) SUV39H1 protein, and (F) p130 protein in *T. scripta elegans* liver. The histogram shows normalized binding levels for protein binding; bold lines represent median values (*n* = 3–4 independent trials on tissue from different animals). Significant differences from the control condition are indicated by ‘a’, while 20 h anoxic values that are significantly different from 5 h anoxia are indicated by ‘b’ (*P* < 0.05).

Changes in the DNA-binding activity of nuclear E2F (E2F1 and E2F4) in response to anoxia were assessed using a transcription factor binding assay and are presented in Fig. 5A. In the liver, the DNA-binding activity of E2F1 did not significantly change in 5 hr of anoxia, however, decreased by 2.4 ± 0.4 - fold in response to 20 hr of anoxia (*P* < 0.05), when compared with control values. Conversely, liver E2F4 showed 1.7 ± 0.1 and 2.6 ± 0.3 - fold increases in binding activity in response to 5 or 20 hr of anoxia, respectively (*P* < 0.05).

Formation of primary DNA-binding E2F complexes in response to anoxia was assessed by detecting proteins known to bind E2F transcription factors. These complex proteins include Rb and p130, both of which binding directly to E2F transcription factors, allowing the assessment primary complex formation (Fig. 5B). In the liver, the E2F association of Rb was increased by 2.5 ± 0.2 - fold in response to 20 hr of anoxia (*P* < 0.05), compared with controls. Binding of p130 did not show any significant differences between control and stress conditions.

The formation of secondary DNA-binding complexes in response to anoxia was assessed by detecting proteins known to bind E2F-bound Retinoblastoma family members (Rb and p130). These secondary complex proteins include HDAC4 and Suv39H1, both of which binding directly to Rb and p130, allowing the assessment secondary complex formation (Fig. 5B). The secondary-binding activity of HDAC4 was increased by 3.9 ± 0.4 - fold in response to 5 hr of anoxia (*P* < 0.05) and by 2.9 ± 0.6 fold in response to 20 hr of anoxia (*P* < 0.05), compared with controls. Suv39H1 showed no significant change after 5 hr anoxia, however, 20 hr anoxia displayed an increase of 1.5 ± 0.1 fold increase, compared to control values (*P* < 0.05).

## Discussion

The intracellular ionic strength of mammalian cells has been reported to be between the values of 110 mM and 250 mM (Motais, Guizouarn & Garcia-Romeu, 1991; Mouat & Manchester, 1998). Optimization of the *in vitro* binding conditions is critical to ensure the proper complex formation and prevention of random protein aggregation with DNA capture probes. These optimization strategies ensure important changes in transcription factor binding, and associated complex members, are not overlooked. This also ensures the elimination of non-specific binding and, of course, that the ionic strength of the DNA binding buffer promotes DNA binding of the transcription factor while preventing dissociation of associated protein complexes. For the binding of E2F and its associated complex partners (Rb, p130, HDAC4, and Suv39H1), an optimal buffer was determined to have an ionic strength of 140 mM and a non-ionic detergent (NP-40) concentration of 0.05% (Supplemental Information). These conditions displayed both maximal binding of the transcription factor, as well as the binding of primary and secondary complex members while minimizing all non-specific interactions between non-specific proteins and complexed proteins, DNA or plastic ware.

The use of co-IP protocols in the evaluation of E2F complexes fail to properly assess the state of cellular proliferation as complexes exist in a free non-DNA bound state. The IP of these individual proteins, or those bound in complex, ultimately yield misleading results as precipitated protein would include non-complexes, free proteins that do not have an influence on transcriptional activity. A typical analysis of active transcription factor complexes also utilizes chromatin IP; the cross-linking of proteins to each other and to the DNA at which it sits (Giovanni, Du & Orr-Weaver, 2001). This technique, although suitable for well-characterized animal models, is time-consuming and extremely difficult to validate for unsequenced animals. The inability to determine the DNA sequence to which its bound (determination of DNA binding site with no genomic information available) and the ability of this technique to generate a high degree of false positives (the crosslinking of near-neighbor proteins, not part of the target complex) makes chromatin IP and costly alternative to the outlined method of DNA-binding transcription factor complex ELISA.

Analysis of the E2F DNA binding complex in the anoxic turtle, using the outlined DNA-binding transcription factor complex ELISA technique, displayed a differential expression of complex members during anoxia. Custom DNA probes were designed and tested specifically for use in *T. scripta elegans*. The ability of turtle-specific E2F to binding this DNA probe (containing the E2F DNA binding sequence) was qualitatively visualized through the use of an EMSA. Banding patterns were similar between turtle proteins and mammalian controls (Fig. 2). For additional validation of probe specificity, a competition assay displayed both reduced DNA binding with the addition of 100-molar excess unlabeled probe, and partial restoration of detectable binding when the competitive probe was mutated within the binding sequence (Fig. 4).

Upon entry into the anoxic state, a switch from active (E2F1) to repressive (E2F4) protein was evident in the liver tissue of anoxic turtles. E2F1 decreased 2.4 ± 0.4 - fold while E2F4 increased 2.6 ± 0.3 –fold in response to 20 hr of anoxia, indicating what would seem to be a complete switch to repressive E2F bound to DNA (Fig. 5A). Also, secondary binding proteins (proteins bound to the Rb-E2F complex) displayed an increase in HDAC4 and Suv39H1 changing 2.9 ± 0.6 and 1.5 ± 0.2 –fold respectively after 20 hr anoxia (Fig. 5B). This result could be indicative of local DNA condensation in the turtle. The possibility of DNA condensation would make sense, due to the proximately of the E2F DNA binding site to genes involved with DNA replication (PCNA, Cyclin A, Geminin, Cdt1), a suppression of the cell cycle through G_1_ arrest could occur in response to hypometabolism and has been previously reported (Bracken et al., 2004; Biggar & Storey, 2009; Roufayel, Biggar & Storey, 2011; Biggar & Storey, 2012). Of particular interest is the binding of either Rb or p130 to the E2F transcription factor, as this is the mechanism widely understood to be the critical different between G_1_ and G_0_ arrest. Similar to other models of anoxia-induced cell cycle arrest, the turtle displayed an increase in Rb complex formation, increasing 2.5 ± 0.3 - fold after 20 hr anoxia, while no significant complex formation was detected for p130 (Fig. 5B).

## Conclusion

Given these results of the study, it would seem likely that the liver tissue of anoxic turtles may enter into a state of G_1_ arrest until normoxia is restored. Indeed, this would suggest conserved low oxygen responsive that is a similar response to that seen in other hypoxia-tolerant organisms, such as *C. elegans* and *D. melanogaster* (Kops et al., 2002; Douglas & Haddad, 2003). Indeed the suppression of cellular proliferation, in part directed by Rb and E2F complex regulation, is likely an important component of overall cellular ATP conservation that is established throughout oxygen deprivation until normoxia can be restored. However, it is important to highlight that a lack of natural chromosomal complexity present in this assay precludes any conclusions about actual transcriptional factor function *in vivo*, and data should be interpreted in this light.

##  Supplemental Information

10.7717/peerj.4755/supp-1 Supplemental Information 1 All raw data for figures Data are absorbance values normalized to control levels. Data are represented as relative fold-change.

10.7717/peerj.4755/supp-2 Supplemental Information 2 Supplemental material documenting protocol optimization Description of methods and optimization figures characterizing the reduction of non-specific protein interaction.

10.7717/peerj.4755/supp-3 Supplemental Information 3 Original blot for EMSA gel. This is the raw image file for Fig. 2 Electromobility shift assay (EMSA) for the E2F family of transcription factors. Distinct bands, as detected with streptavidin HRP, is indicative of E2F binding as detected in control, 5 h and 20 h anoxic turtle liver tissues, as well as the positive control. Negative control lanes include (1) turtle liver proteins without DNA probe and (2) DNA probe without any protein. Bands at the bottom of the gel are indicative of single (incompletely annealed) and double stranded biotinylated DNA probe.

## Additional Information and Declarations

### Data Availability

The following information was supplied regarding data availability: The raw data are provided in the Supplemental Files.
