# Supplementary material for: The evaluation of anoxia responsive E2F DNA binding activity in the red eared slider turtle, Trachemys scripta elegans
Source: PeerJ. 2018 May 11;6:e4755. doi: 10.7717/peerj.4755 (PMC5951122; doi:10.7717/peerj.4755)
Supplement: Supplemental Information 2 — Description of methods and optimization figures characterizing the reduction of non-specific protein interaction. [file peerj-06-4755-s002.docx]

**SUPPLEMENTARY MATERIAL**

**Effect of ionic strength on protein binding**

To determine the ionic strength necessary for maximal binding of complex members, while minimizing the binding of non-specific nuclear proteins, DNA-binding transcription factor complex ELISAs were carried out at several ionic strength conditions approximate to physiological conditions. To determine the optimal ionic strength for DNA-binding complex formation, the presence of both E2F1 and Rb were detected at several ionic strengths (Fig.1S). All buffer conditions were tested with a low concentration of non-ionic detergent (0.05% NP-40). In the liver, the E2F1 significantly bound to the DNA probe under all ionic strength condition, however, maximal binding was seen at an ionic strengths of 100 mM (P<0.05) and 140 mM (P<0.05), when compared to 80 mM ionic strength. Complex formation of Rb was also highest at an ionic strength near physiologically relevant conditions of 100 mM (P<0.05) and 140 mM (P<0.05), when compared to 80 mM ionic strength. The specificity of the DNA-binding transcription factor complex ELISA was determined by attempting to detect a transcription factor (XBP1) bound to the E2F DNA probe. The non-specific binding of XBP1 was detectable below physiological ionic strength (< 100 mM) and decreased significantly above an ionic strength of 140 mM (P<0.05), compared to both 80 and 100 mM values (Fig.1S).

**Effect of non-ionic detergent concentration on binding**

To determine the non-ionic detergent strength necessary for maximal binding of complex members, while preventing the binding of non-specific nuclear proteins, DNA-binding transcription factor complex ELISAs were carried out at several detergent concentrations in either binding buffers of 100 mM or 140 mM ionic strength. Binding buffers were selected based on their ability to maximize DNA binding and complex formation, while preventing non-specific binding (Fig.1S). To determine the optimal strength for E2F-Rb complex formation, the presence of both E2F1 and Rb were detected at several concentrations of NP-40 (Fig.2S). E2F1 significantly bound to the DNA probe under most non-ionic detergent conditions. No significant changes in E2F1 binding were found irrespective of detergent concentration or buffer ionic strength; however, a significant reduction of E2F1 binding was seen at 100 mM ionic strength in an absence of NP-40 (P<0.05). Additionally, E2F1 binding decreased significantly at 0.5% NP-40 in 140 mM ionic strength buffer, compared to 0% NP-40 values (P<0.05). Primary complex formation of Rb was highest at a detergent concentration of 0.05%-0.5% NP-40 at 100 mM and 0.05% at 140 mM (P<0.05), compared to 0% NP-40 values. Binding of XBP1 was only detectable between 0-0.05% NP-40 at 100 mM ionic strength and 0% NP-40 at 140 mM ionic strength when compared to all other values (P<0.05) (Fig.2S). As a result of the low non-specific binding of XBP1 and high binding of both E2F and Rb, a binding buffer of 140 mM and 0.05% NP-40 was chosen for all further quantification experiments.


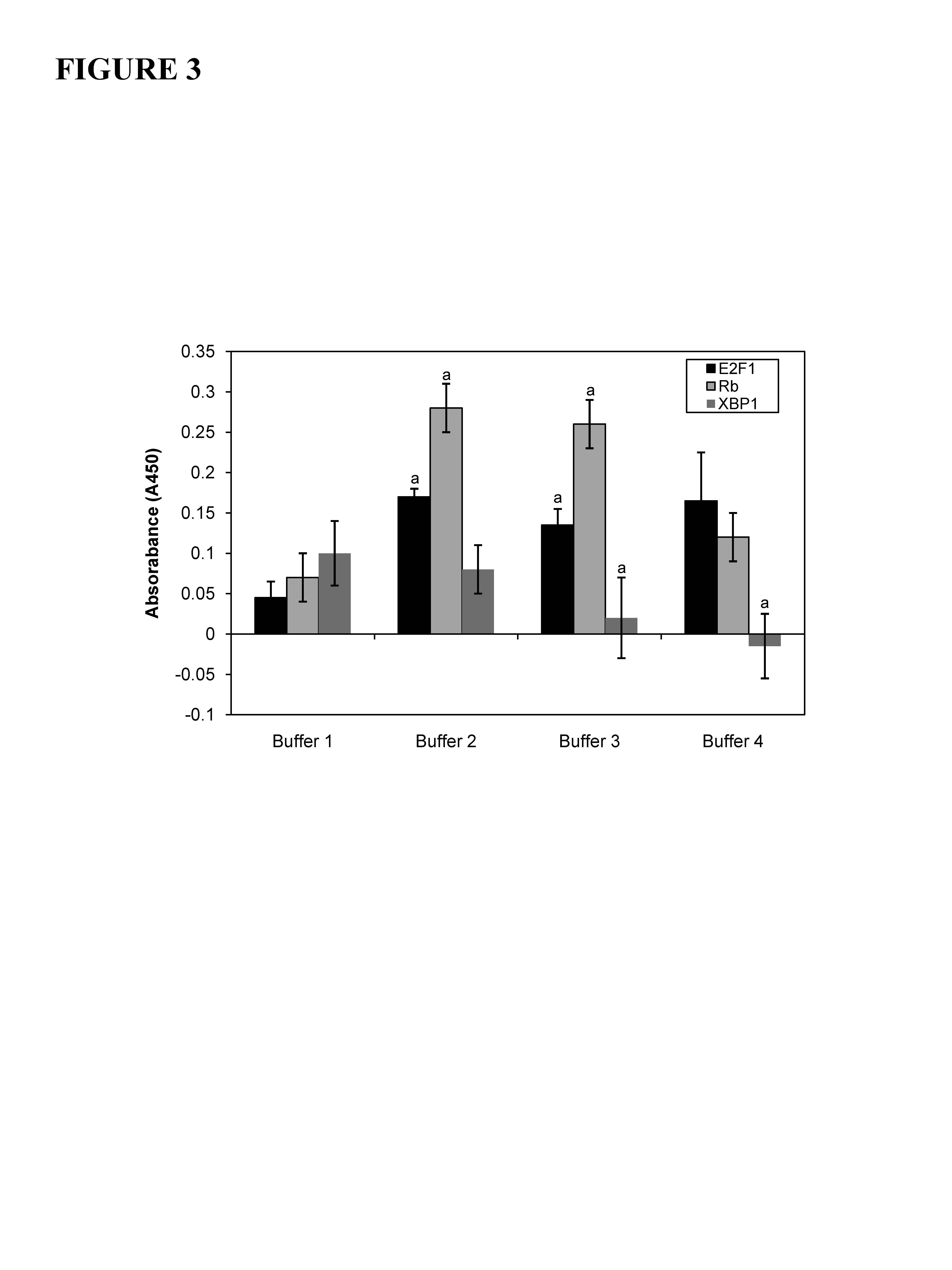


FigS1. Effect of ionic strength on the relative binding of E2F1, Rb and XBP-1 (non-specific) in liver from control *T. scripta elegans*. Ionic strengths for representative buffers are; Buffer 1 (80 mM), Buffer 2 (100 mM), Buffer 3 (140 mM) and Buffer 4 (220 mM). Histogram shows normalized binding levels for various buffer ionic strengths and the influence on protein binding; data are means ± SEM. (*n* = 3–4 independent trials on tissue from different animals). Significant differences from the 80 mM condition are indicated by 'a' (*P*< 0.05).


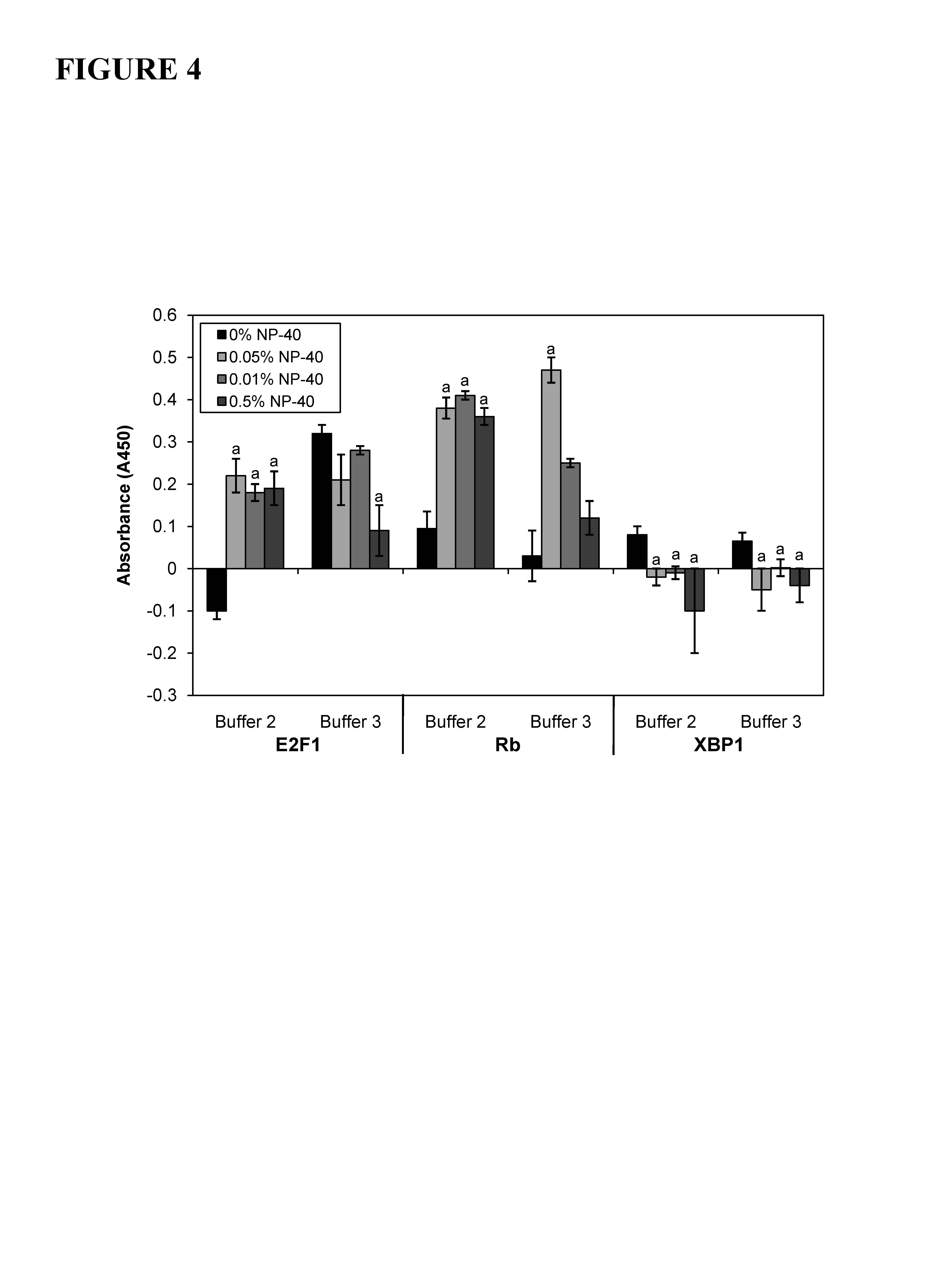


FigS2. Effect of non-ionic detergent concentration (varied by % NP-40) on the relative binding of E2F1, Rb and XBP-1 (non-specific) in liver from control *T. scripta elegans*. Non-ionic detergent concentration was varied in two different ionic strength buffers; Buffer 2 (100 mM) and Buffer 3 (140 mM). Histogram shows normalized binding levels for various non-ionic detergent concentrations and its influence on protein binding; data are means ± SEM. (*n* = 3–4 independent trials on tissue from different animals). An 'a' indicates significant differences from the 0% NP-40 concentration in the respective buffer (*P*< 0.05).

**SUPPLEMENTARY TABLE**

**Table S1.** Assay conditions for determining presence in E2F DNA-bound protein complex.

| **Target** | **Nuclear protein (µg)** | **Protein incubation (h)** | **Primary antibody dilution; Incubation (h)** | **Secondary antibody dilution; Incubation (h)** |
| --- | --- | --- | --- | --- |
| anti-E2F1 rabbit | 16 | 1 | 1:1000; 1 | 1:2000; 1 |
| anti-E2F4 rabbit | 16 | 1 | 1:1000; 1 | 1:2000; 1 |
| anti-Rb mouse | 16 | 1.5 | 1:1000; 1.5 | 1:1000; 1 |
| anti-p130 goat | 24 | 1.5 | 1:500; 1.5 | 1:4000; 1 |
| anti-Suv39H1 goat | 16 | 1.5 | 1:1000; 1.5 | 1:4000; 1 |
| anti-HDAC4 mouse | 16 | 1.5 | 1:1000; 1 | 1:1000; 1 |
| anti-XBP1 rabbit | 16 | 1.5 | 1:1000; 1.5 | 1:1000; 1 |
