# Supplementary figures and images for: The evaluation of anoxia responsive E2F DNA binding activity in the red eared slider turtle, Trachemys scripta elegans
Source: PeerJ. 2018 May 11;6:e4755. doi: 10.7717/peerj.4755 (PMC5951122; doi:10.7717/peerj.4755)

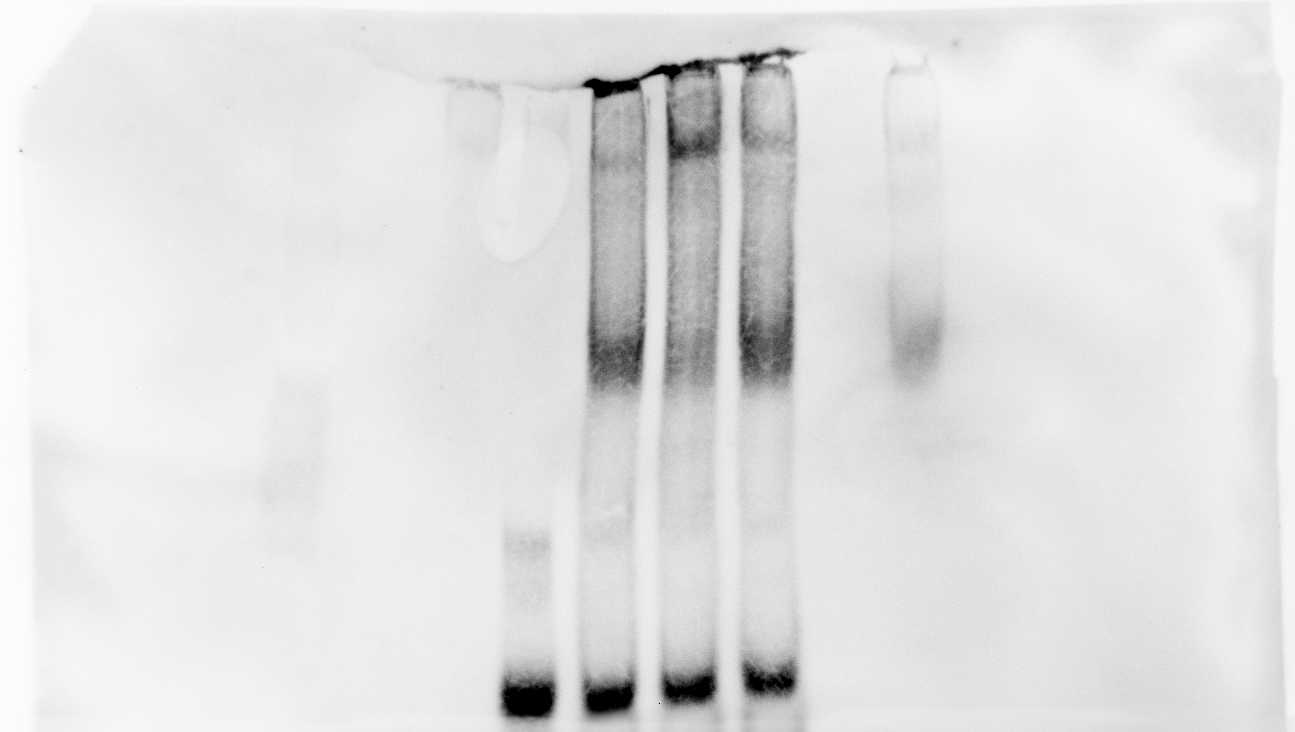

Supplement: Supplemental Information 3 — Electromobility shift assay (EMSA) for the E2F family of transcription factors. Distinct bands, as detected with streptavidin HRP, is indicative of E2F binding as detected in control, 5 h and 20 h anoxic turtle liver tissues, as well as the positive control. Negative control lanes include (1) turtle liver proteins without DNA probe and (2) DNA probe without any protein. Bands at the bottom of the gel are indicative of single (incompletely annealed) and double stranded biotinylated DNA probe. [file peerj-06-4755-s003.jpg]
